# Stereotactic body radiation therapy in primary liver tumor: Local control, outcomes and toxicities

**DOI:** 10.1016/j.ctro.2024.100892

**Published:** 2024-11-21

**Authors:** Ludovic Hernandez, Laure Parent, Victoire Molinier, Bertrand Suc, Françoise Izar, Elisabeth Moyal, Jean-Marie Peron, Philippe Otal, Amélie Lusque, Anouchka Modesto

**Affiliations:** a Department of Radiotherapy, University Cancer Institute of Toulouse-Oncopole, 31100 Toulouse, France; b Department of Digestive Surgery and Liver Transplantation, Rangueil University Hospital, 31059 Toulouse, France; c Department of Hepatogastroenterology, Rangueil University Hospital, 31059 Toulouse, France; d Department of Radiology, Rangueil University Hospital, 31059 Toulouse, France; e Biostatistics Unit, University Cancer Institute of Toulouse-Oncopole, 31100 Toulouse, France

**Keywords:** Stereotactic body radiation therapy, Hepatocellular carcinoma, Efficacy, Safety, Local Control

## Abstract

**Objective:**

Stereotactic body radiation therapy (SBRT) is a therapeutic option in the guidelines for liver primaries after standard strategies like surgery or thermoablation have failed. To assess its efficacy and safety, we reviewed all patients treated by SBRT for a hepatocellular carcinoma (HCC) over a six-year period.

**Methods and materials:**

The study included all patients treated by SBRT for HCC between April 2015 and November 2021 in the University Cancer Institute at Toulouse-Oncopole. All patients were inoperable and not eligible for thermoablation, or after a failure. All tumor sizes were included and cirrhosis up to Child-Pugh B was accepted. Local control (LC), overall survival (OS) and progression-free survival (PFS) were estimated by the Kaplan-Meier method. Treatment response was assessed using mRECIST criteria. Toxicity was graded using CTCAE (v4.0).

**Results:**

One hundred and nine patients with 118 lesions were treated. Half underwent prior standard treatment. Median dose was 50 Grays in five fractions for most patients. Chronic liver disease represented 90.8 % of cases with a median age of 69 years. Median tumor size was 4.0 cm. Median follow-up was 22.2 months [95 %CI: 15.1–30.4]. LC, OS and PFS at two years were 82.4 % [95 %CI: 71.3; 89.5], 73.2 % [95 %CI: 61.5; 81.8] and 35.8 % [95 %CI: 25.1; 46.7], respectively. Acute toxicities occurred in 20.2 % of patients, including 10.1 % grade 3–4 and 1.8 % grade 5. Late toxicities occurred in 5.5 % of patients including 4.6 % grade 3–4. Grade ≥ 3 toxicity was related to digestive perforation or liver failure.

**Conclusion:**

SBRT provides good LC with an acceptable safety profile. It can be used in several settings such as salvage therapy or in combination with validated treatment. Prospective randomized trials are needed to validate SBRT as a standard alternative.

## Highlights

• Stereotactic body radiation therapy is a curative alternative for HCC.
• Efficacy and safety of SBRT appear to be the same as those of thermoablation or surgery.
• SBRT can be used as salvage therapy or in combination with standard treatment.
• Prospective randomized studies are needed to define role of SBRT.

## Introduction

Liver cancer is the sixth most common malignant disease and the third cause of cancer-related death worldwide [1]. Hepatocellular carcinoma (HCC) accounts for approximately 90 % of liver malignancies at diagnosis. HCC usually develops from preexisting chronic liver disease, such as cirrhosis due to alcohol abuse or non-alcoholic steatohepatitis (NASH), chronic viral hepatitis C or B (HBV or HBC), or metabolic disorders of variable pathogenesis [2]. Tumor management should therefore seek to spare liver function. Treatment decisions are usually based on tumor staging, liver function assessment, and performance status, especially in Europe and North America [3], [4], using the Barcelona Clinic Liver Cancer (BCLC) staging [5].

Surgery is the standard curative treatment in the early stages of liver cancer, with five-year overall survival (OS) ranging from 50 to 70 % [2], [6], [7]. However, only approximately 30 % of patients are able to receive surgery and fewer than 20 % receive liver transplantations [8]. Thermoablation (TA), such as radiofrequency ablation (RFA) and microwave ablation, offer validated alternatives to surgery. For tumors ≤ 3 cm, it provides comparable efficacy to that of surgical resection in terms of OS and progression-free survival (PFS) with a relapse rate of approximately 60 % and excellent local control (LC) rates from 70 % to 90 % [9], [10]. However, these results concern only the early stages, as these ablative techniques are less efficient for tumors > 5 cm or those located the near major vessels, heart, diaphragm or bile ducts [11].

Patients with intermediate-stage tumors who cannot attend either surgery or receive TA can be treated with transarterial therapies such as transcatheter arterial chemoembolization (TACE), which has shown promising LC rates around 52 %. Nevertheless, survival rates remain low with three-year OS and PFS of 40 % and 15 %, respectively [12].

Stereotactic body radiation therapy (SBRT) is also a promising therapeutic option. Among the local therapeutic strategies, radiotherapy has long been considered as a palliative option [13]. External beam conformational radiation therapy is excessively toxic, potentially causing radiation-induced liver disease (RILD) [14]. SBRT is a recent technique that delivers high doses of radiation to cancer cells in a limited number of fractions, while reducing damage to healthy tissue thanks to precise positioning. Several uncontrolled studies have demonstrated the potential benefit of SBRT in liver cancer management, alone or in association with another local therapy, showing similar tumor control rates in comparison to standard ablative techniques [15], [16], [17], [18]. Furthermore, a significant heterogeneity exists in practices with variations in fractionation schemes depending on the continent. Recent recommendations suggest that biologically effective dose assuming an α/β = 10 (BED_10_) should be greater than 75 Gy to achieve a curative effect [19], [20].

Even though numerous publications support the use of SBRT in these indications, more real-life data are needed while waiting for prospective studies. This study retrospectively analyzed all patients treated by SBRT for a HCC, in the Department of Radiation Oncology at the University Cancer Institute of Toulouse − Oncopole, in order to evaluate its efficiency and safety in a homogeneously treated population.

## Materials and methods

### 1 Data collection

All patients receiving hepatic SBRT as treatment for HCC between April 2015 and November 2021 in the Department of Radiation Oncology at the University Cancer Institute of Toulouse − Oncopole, were retrospectively analyzed. All included patients were inoperable and/or unresectable and not eligible to TA (due to the patient's condition, tumor size >3 cm and/or location), or had experienced failure with this method. SBRT could be performed as part of a multimodal treatment in combination with TACE or as a bridging treatment while awaiting liver transplantation. All tumor sizes and disease stages according to the BCLC classification were included. Exclusion criteria were the presence of decompensated cirrhosis (>Child-Pugh B7 score still eligible), an ascites, functional liver parenchyma <1000 ml, an ECOG-PS >2, non-cooperative patients, or a radiosensitizing concurrent treatment. Previous treatment with other local modalities was not an exclusion criterion. Diagnosis of HCC was established by either radiological criteria (defined as the detection of contrast hyper-enhancement in the arterial phase (wash-in) and hypo-enhancement in the portal or delayed phase (wash-out) by computed tomography (CT) scan or magnetic resonance imaging (MRI)), or histopathological findings. A complete work-up using multi-phase CT scan or MRI was performed for each patient. The indication for SBRT was validated systematically during a regional multidisciplinary liver tumor board meeting including surgeon, radiologist, gastro-oncologist and radiation oncologist. The study was approved by the local institutional boards and was registered in Health Data Hub (F20220808094532).

## 2 Radiotherapy planning

According to local standards, three fiducial markers (FM) were placed ideally within 5 cm of the tumor one week before the radiotherapy planning CT scan, unless patients already had radiopaque markers from a former intervention, such as surgical clips or a transjugular intrahepatic portosystemic shunt. The placement of FM was performed by an experienced radiologist using CT or ultrasound guidance and under local anesthetic. Multiphase four-dimensional contrast enhanced computed tomography (4D-CT) was performed in the treatment position to incorporate information on respiratory motion into the treatment planning process, with the patient supine and fasting for a minimum of four hours.

Orfit’s immobilization and positioning system with four-points thoracic masks was performed for each patient. MRI was performed in the treatment position promptly after 4D-CT in order to undertake CT-MRI image fusion.

The 4D-CT was divided into six breathing phases and the gross tumor volume (GTV) was defined on the expiratory phase (corresponding to the 50 % breathing cycle images), with an MRI-matching process in treatment position, including early contrast enhancement and late washout. Contouring was performed with the help of experienced radiologists. The planning target volume (PTV) contained GTV with a 0.5 cm margin in all directions and a 0.8 cm margin in the Superior-Inferior (SI) direction. The organs at risk (OAR) located within 5 cm of the PTV in the SI direction were contoured: liver, lungs, aorta, vena cava, esophagus, stomach, duodenum, small and large intestines, gall bladder, kidneys, chest wall and medullar canal.

The usual irradiation schemes were 45–54 Grays (Gy) in three fractions or 45–50-55 Gy in five fractions based on 80 % of the prescribed isodose surfaces. The fractionation scheme was defined by PTV proximity with OARs. If PTV distance was >5 mm from the OARs and meets the constraints for the three-fraction regimen, three fractions of either 15 or 18 Gy could be prescribed (BED_10_ = 112.5 or 151.2 Gy, respectively). For PTVs with extensive contact with the chest wall or the aorta/vena cava, five fractions of 10 to 11 Gy could be prescribed (BED_10_ = 100 to 115.5 Gy). In case of PTVs close to OARs (particularly digestive tract), 45 to 50 Gy delivered in five fractions were prescribed (BED_10_ = 85.5 or 100 Gy, respectively). All patients received alternate day treatment. OAR constraints following standard guidelines evolved over the course of the study and the most recent ones are summarized in Table 1. Constraints on Liver-GTV, Gallbladder/bile ducts, and Digestive tract were strictly applied. All dosimetric results were evaluated based on dose-volume histograms. Data were collected in Aria® (Varian) using DoseHunter, an open-source script.

**Table 1:** **Dose constraints to organs at risk according to SABR UK consortium (V6,1–2019)**.

Fraction number	3	5
Heart	Dmax_(0.5cc)_ < 26 Gy	Dmax_(0.5cc)_ < 29 Gy
		V_32Gy_ < 15 cc
		
Aorta/Vena Cava	Dmax_(0.5cc)_ < 45 Gy	Dmax_(0.5cc)_ < 53 Gy
		
Lungs	D_(≥1500cc)_ ≤ 10.5 Gy	D_(≥1500cc)_ ≤ 12.5 Gy
	D_(≥1000cc)_ ≤ 11.4 Gy	D_(≥1000cc)_ ≤ 13.5 Gy
		
Chest Wall	Dmax _(0.5cc)_ < 37 Gy	Dmax _(0.5cc)_ < 39 Gy
	V_30Gy_ < 30 cm^3^	V_32Gy_ < 30 cm^3^
		
Duodenum	Dmax_(0.5cc)_ < 22.2 Gy	Dmax_(0.5cc)_ < 35 Gy
	V_16.5Gy_ < 5 cm^3^	V_25Gy_ < 10 cm^3^
	V_11.4Gy_ < 10 cm^3^	
		
Stomach	Dmax_(0.5cc)_ < 22.2 Gy	Dmax_(0.5cc)_ < 35 Gy
	V_16.5Gy_ < 10 cm^3^	V_25Gy_ < 10 cm^3^
		
Small intestines	Dmax_(0.5cc)_ < 25.2 Gy	Dmax_(0.5cc)_ < 35 Gy
	V_17.7Gy_ < 5 cm^3^	V_25Gy_ < 10 cm^3^
		
Large intestines	Dmax _(0.5cc)_ < 28.2 Gy	Dmax _(0.5cc)_ < 32 Gy
		
Liver-GTV	D_(≥700cc)_ ≤ 19,2 Gy	D_(≥700cc)_ ≤ 21 Gy
		Dmean < 15,2 Gy
		
Kidney	D_(≥200 cc)_ ≤ 15 Gy	V_10Gy_ < 45 %
		D_(≥ 200cc)_ ≤ 17,5Gy
		Dmean < 10 Gy
		
Medullar Canal	Dmax_(0.1cc)_ < 22 Gy	Dmax _(0.1cc)_ < 30 Gy
	V_18Gy_ < 0.35 cc	V_22.5Gy_ < 0.35 cc
		
Gallbladder	Dmax_(0.5 cc)_ < 50 Gy	Dmax_(0.5 cc)_ < 50 Gy
		

Radiation therapy was performed using dynamic-modulated arc therapy with flattening filter free 10 MV (MegaVolt) beams on a Novalis True Beam accelerator. Respiratory movement was recorded by an external surrogate placed on the patient’s abdominal wall. Real-time monitoring of FM using fluoroscopy was performed during treatment. The treatment was delivered in the 33 % to 66 % range of the breathing cycle, corresponding to the expiratory phase, using the gating technique.

The calculation of dose equivalency was based on a linear quadratic model. Given the different irradiation schemes used, the dose received was converted into a BED calculated according to equation (1) with d, the number of fractions, D the radiation dose, and α/β = 10.

(1) $${\text{BED}}_{\alpha /\beta }=D\;x\left(1+\left[d/\left(\alpha /\beta \right)\right]\right)$$

## 3 Follow-up

Follow-up included clinical and radiological examination three months after SBRT completion and then every three months by multiphase CT or MRI. All images were reviewed centrally by an expert radiologist during a regional liver tumor board meeting. Treatment response was assessed according to mRECIST criteria, defined as progression (P), stable disease (SD), partial (PR) and complete response (CR).

Toxicities were collected retrospectively from medical files and graded using CTCAE NCI-CTC Version 4.0. An early adverse effect was defined as any event occurring within six months from the end of treatment, and a late adverse effect as any event occurring thereafter. Status was determined by the Eastern Cooperative Oncology Group − Performance Status (ECOG PS). Radiation-induced liver disease (RILD), which is a serious complication of radiation therapy (RT), was defined as anicteric hepatomegaly, ascites, and elevated liver enzymes, especially alkaline phosphatase, occurring typically four to eight weeks after completion of RT, and as late as 12 months. Non-classical RILD concerned patients with underlying liver diseases, with prior jaundice, elevated serum transaminase (grade III-IV), a high Child-Pugh score (8 or more: decompensated cirrhosis), or profound thrombocytopenia.

## 4 Statistical analysis

Data were summarized by frequency and percentage for qualitative variables and by median and range for continuous variables. OS was defined as the time from treatment initiation to death or to the last follow-up (censored data). PFS was defined as the time from treatment initiation to progression or death. Patients still alive and without progression were censored at the last follow-up. LC was defined as the time from treatment initiation to local progression or to the last follow-up (censored data). All survival times were estimated by the Kaplan-Meier method with 95 % confidence intervals (CI). Univariable analyses were performed using the Log-rank test for qualitative variables and the Cox proportional hazards model for continuous variables. All statistical tests were two-sided and p-values < 0.05 were considered significant. Statistical analyses were conducted using Stata®, version 16.

## Results

### 1 Patient and radiotherapy characteristics

**Fig. 1:**
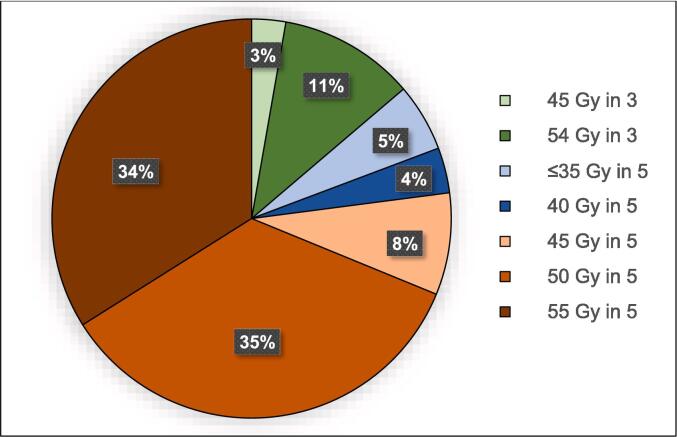
Doses and fractionations in all 109 patients.

From April 2015 to November 2021, we analyzed 109 patients treated by SBRT for HCC with an overall number of 118 HCC. Twenty-four patients were females and 85 were males. Median age at initiation of SBRT was 69 years (range, 37–90). Median follow-up time was 22.2 months [95 %CI: 15.1–30.4]. The causes of cirrhosis were exogenous in 57.8 % of patients, related to NASH in 31.2 %, and due to hepatitis C or B in 20.2 %. Most patients had Child-Pugh A compensated cirrhosis (77.8 %). Regarding BCLC stage, 6.4 % were very early stage (0), 47.7 % initial stage (A), 26.6 % intermediate stage (B) and 19.3 % advanced stage (C). Median tumor diameter and volume were 4.0 cm (range, 1.6–9.0) and 24.9 cm^3^ (range, 1.8–305.0), respectively. Main characteristics of patients and tumors are summarized in Table 2. Median delivered dose was 50 Gy (range, 30–55), in five fractions for 86.2 % of patients or three fractions for 13.8 %. Detailed fractionations and doses are listed in Fig. 1. SBRT was a bridge strategy to transplantation in 11 patients (10.1 %). Median BED_10_ was 100 Gy (range, 48.0–151.2). Prior treatment on target lesion had been performed for 54.1 % of patients; in 47.7 % of cases the treatment included a TACE. Main radiation therapy parameters are shown in Table 3.

**Table 2:** Patient and tumor characteristics at diagnosis. * The values listed in the table give the number of patients (n) followed by the percent of patients (%), unless otherwise specified.

		Value	
Total		109	100 %
Sex	Female	24	22.0%
	Male	85	78.0%
			
Age in years	Median (range)	69 (37–90)	
	≤70	60	55.0 %
	>70	49	45.0 %
			
ECOG PS	0	67	61.5 %
	1	37	33.9 %
	2	5	4.6 %
			
Cirrhosis		99	90.8 %
Etiology of cirrhosis	HCV infection	22	20.2 %
	Alcohol abuse	63	57.8 %
	NASH	34	31.2 %
	Other	7	6.4 %
			
Child-Pugh score	A5	64	59.3 %
	A6	20	18.5 %
	B7	16	14.8 %
	B8	7	6.5 %
	B9	1	0.9 %
	Missing	1	
			
BCLC stage	0	7	6.4 %
	A	52	47.7 %
	B	29	26.6 %
	C	21	19.3 %
			
Diagnosis	Histological	42	38.5 %
	Image-based	67	61.5 %
			
Tumor thrombosis		6	5.5 %
		
Extrahepatic disease		2	1.8 %
		
Liver transplantation waiting list		26	23.9 %
		
Number of tumors	1	100	91.7 %
	2	9	8.3 %
			
Median hepatic volume in cm^3^ (range)		1607.8 (621.5–4213.9)	
	
Tumor size in cm	Median (range)	4.0 (1.6–9.0)	
	<3 cm	20	18.3 %
	3–5 cm	59	54.1 %
	≥5 cm	30	27.5 %
			
Median GTV in cm^3^ (range)		24.9 (1.8–305.0)	
	
Median PTV in cm^3^ (range)		75.0 (16.4–433.2)	
	
Invaded liver segments	**Total**	**134**	**100 %**
	I	6	4.5 %
	II	8	6.0 %
	III	6	4.5 %
	IV	36	26.9 %
	V	17	12.7 %
	VI	12	9.0 %
	VII	18	13.4 %
	VIII	31	23.1 %

**Table 3:** Characteristics of radiation therapy.

		n	%	Median	Range
**Total**		**109**	**100 %**		
Duration of SBRT (days)				11.0	5.0–24.0
			
Total dose in BED_10_ (Gy)	All			50.0	30.0–55.0
	<75 Gy	10	9.2 %		
	≥75 Gy	99	90.8 %		
				
Maximal dose (Gy)				66.3	40.0–75.2
			
D95% (Gy)	Prescribed			52.3	18.7–58.3
	EQD2			87.7	31.2–137.8
	BED_10_			105.2	37.5–165.3
				
Liver mean dose (Gy)	Prescribed			8.5	2.1–21.8
	EQD2			15.0	3.2–34.5
	BED_10_			18.9	3.8–41.4
				
Prescribed dose (Gy)	EQD2			83.3	40.0–126.0
	BED_10_			100.0	48.0–151.2
				
Type of markers	Stent	2	1.8 %		
	Fiducial	97	89.0 %		
	Lipiodol	8	7.3 %		
	Surgical Clips	1	0.9 %		
	Other	1	0.9 %		
				
Prior treatment on target lesion	**Total**	**59**	**54.1 %**		
	TACE	35	32.1 %		
	RFA	6	5.5 %		
	Systemic treatment	2	1.8 %		
	TACE + RFA	13	11.9 %		
	TACE + Systemic	3	2.8 %		
	RFA + Alcohol	1	0.9 %		
	TACE + SBRT	1	0.9 %		
				

### 2 Survival analysis

**Fig. 2:**
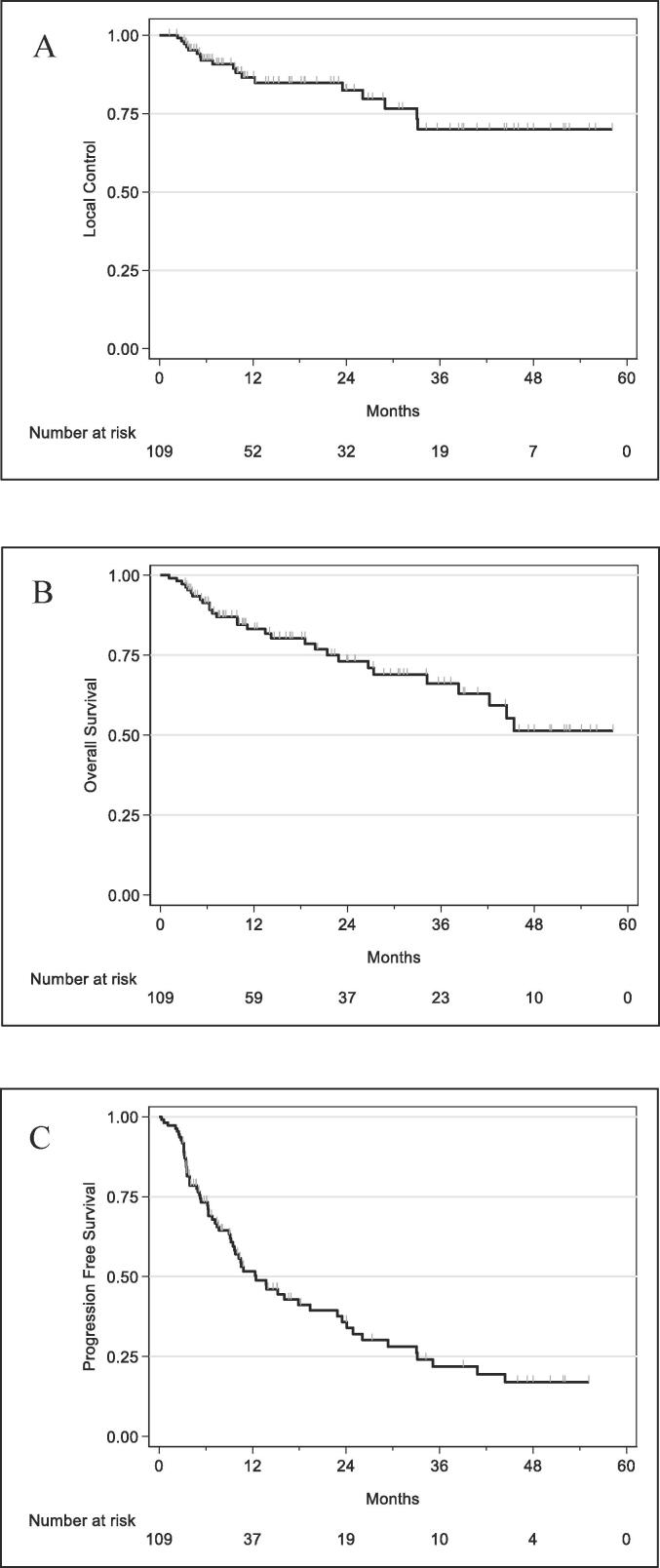
Kaplan-Meier analysis in all 109 patients: Local Control is represented in A, Overall survival (OS) in B, and Progression-Free Survival (PFS) in C.

OS at one and two years was 83.2 % [95 %CI: 73.9–89.4] and 73.2 % [95 %CI: 61.5–81.8] and PFS at one and two years was 51.7 % [95 %CI: 40.9–61.5] and 35.8 % [95 %CI: 25.1–46.7], respectively (Fig. 2). Median PFS was 12.5 months [95 %CI, 9.5–22.9]. Initial maximal size of the lesion was significantly associated with worst PFS in univariable analysis, with an HR of 1.25 (95 % CI, 1.07 to 1.45; p = 0.004). Considering survival in curative patients who received BED_10_ ≥ 75 Gy, OS at one and two years was 83.6 % [95 %CI: 73.6; 90.0] and 74.0 % [95 %CI: 61.5; 83.0], and PFS at one and two years was 53.5 % [95 %CI: 42.0; 63.6] and 37.0 % [95 %CI: 25.4; 48.6], respectively. Initial maximal size of the lesion remained associated with worst PFS with an HR of 1.21 (95 %CI, 1.02 to 1.44; p = 0.028).

### 3 Local control

According to mRECIST criteria, best response was complete in 59.0 %, partial in 20.0 %, stable in 13.3 % and progressive disease in 7.6 %. LC of the irradiated target was 86.5 % [95 %CI: 77.2–92.2] at one year from SBRT and 82.4 % [95 %CI: 71.3–89.5] at two years (Fig. 2). In univariable analysis, the main prognostic factors (sex, ECOG PS, CHILD score, Alpha foetoprotein level, tumor size) were not statistically associated with LC. BED_10_ ≥ 100 Gy is associated with greater LC at 1 year in comparison to BED_10_ < 100 Gy (91.1 % [95 %CI: 81.1;95.9] vs 67.8 % [95 %CI:40.8;84.5]; p = 0.033). In addition, tumor diameter ≥ 3 cm in comparison to <3 cm (83.6 % [57.3;94.4] vs 87.4 % [76.8;93.3]; p = 0.9533), prior treatments by TACE compared to no TACE (85.7 % [95 %CI: 72.0;93.0] vs 87.9 % [95 %CI: 72.6;94.9]; p = 0.591) or TA compared to no TA (84.8 % [95 %CI: 73.9;91.4] vs 94.7 % [95 %CI: 68.1;99.2]; p = 0.553) were not significantly associated with better LC at 1 year. Similar results were found in the 99 patients receiving SBRT with BED_10_ ≥ 75 Gy (90.8 % of all), considered curative care: LC at one and two years was 88.8 % [95 %CI: 79.5; 94.1] and 84.1 % [95 %CI: 72.1; 91.3], respectively. Regarding tumor control, there was no other significant factor in this specific group of patients.

### 4 Adverse events

Immediate tolerance of the treatment was acceptable, with a total of reported acute toxicities of 20.2 % for all stage tumors, including 10.1 % of patients presenting severe grade 3–4 toxicities. Two patients died within three months after SBRT (1.8 %) from acute liver failure. One of them was an 81-year-old woman with compensated cirrhosis, Child-Pugh A5, associated with NASH and a 3.1 cm tumor. She had been treated by TA on another target one year before receiving SBRT. She received 54 Gy in 3 fractions, with an acceptable mean dose to uninvolved liver of 4.4 Gy. The second was a 71-year-old patient with Child-Pugh B7 cirrhosis associated with hemochromatosis. and a 4.8 cm lesion. He received 50 Gy in 5 fractions, with a mean Liver-GTV dose of 15 Gy. He had been treated by TACE on the target lesion, 3 months before SBRT. Late adverse events (6 months after RT completion) occurred in 5.5 % of patients: 0.9 % had grade 1–2 toxicities and 4.6 % had grade 3–4 toxicities: mainly digestive tracts ulceration despite respect to dose constraints. A complete baseline or post-treatment biological assessment was available for 45 % of the patients. The main adverse event data are summarized in Table 4.

**Table 4:** Early and late adverse events occurring during and after SBRT.

		Total		Grade max				
		n	%	1	2	3	4	5
**Acute Toxicities**	**Number of patients**	**22**	**20.2**					
	Parietal pain	9	8.3	4	5			
	Nausea	9	8.3	6	2	1		
	Abdominal pain	6	5.5	3	3			
	Hepatic decompensation	5	4.6		2	2	1	
	Diarrhea	4	3.7	2	2			
	Non-classical RILD	4	3.7	3	1			
	Fatigue	3	2.8	1	2			
	Gastric ulcer	2	1.8		1	1		
	Hepatic failure	2	1.8					2
	Cholecystitis	2	1.8			2	2	
	Gastric perforation	1	0.9			1		
	Flatulence	1	0.9	0	1			
	Rib Fracture	1	0.9		1			
	Duodenal fistula	1	0.9			1		
								
**Late Toxicities**	**Number of patients**	**6**	**5.5**					
	Gastric ulcer	2	1.8			2		
	Duodenal ulcer	2	1.8			2		
	Hepatic Abscess	1	0.9			1		
	Pleural effusion	1	0.9	1				

## Discussion

While SBRT represents a curative alternative for hepatocellular carcinoma, its recentness precludes its indication as a first line option. As the management of HCC remains challenging, we retrospectively analyzed data from unselected consecutive patients homogeneously treated by SBRT for HCC. After a median follow-up of two years, LC at one and two years was 86.5 % and 82.4 % respectively, in the whole sample (n = 109). The annex shows similar series of patients treated by SBRT. Ours is one of the largest retrospective series treated in homogeneous conditions in recent years.

The difference in LC in our series may be explained by several factors. Firstly, median tumor size was 4.0 cm (range, 1.6–9.0), unlike in most prior studies in which patients had smaller HCC lesions with a median size of 1.7 to 3 cm [15], [16], [17], [18], [21], [22], [23]. In series focusing on larger tumors measuring around 5 cm with locally advanced disease, LC was approximately 85 % [24], [25], [26], which is similar to our results. Interestingly, we did not find any significant association between tumor size and LC rate (<3 cm vs ≥ 3 cm, 83.6 % [57.3;94.4] vs 87.4 % [76.8;93.3], respectively; p = 0.9533), probably due to larger tumor sizes in our series (54.1 % from 3 to 5 cm, and 27.5 % ≥ 5 cm). Furthermore, these lesions had often already been pre-treated (54.1 %) or were localized in complex anatomical positions (26.9 % in segment IV and 23.1 % in segment VIII, often nearby the heart or hollow organs which may have impact target coverage to protect OARs). Tumor size is known to be prognostic in the management of primary liver tumors, especially when the lesion is < 3 cm in size [18], [26].

Underlying liver disease profiles were the second main reason for our differing results. Most of our patients suffered from exogenous cirrhosis (57.8 %) or metabolic NASH (31.2 %), with fewer virally induced cancers (20.2 %). Many reports to date were based on Asian populations with between 65 % and 88 % patients developing oncogenesis on viral hepatitis [16], [23], [24], [26]. Moreover, liver function tended to be better in prior studies, with higher rates of Child-Pugh A liver diseases (85 % on average, range 76 % − 91 %) [16], [18], [23], [24], contrary to the 77.8 % rate of low grade cirrhosis in our cohort. Consequently, patient profiles were different, with a larger number of patients with serious comorbidities such as persistent dysmetabolic syndromes or alcohol use and extended diseases in our series. Our data were retrieved from unselected patients, as in some prospective studies, which is consistent with standard clinical practice.

Recent metaanalyses all agreed on the relevance of SBRT in liver tumor management. The two main metanalyses, which were essentially retrospective, focused on small (mean size 2.3–2.9 cm) early-stage HCC. They reported LC rates of around 95 % at one year and over 90 % at three years [27], [28]. In another less selective retrospective metanalysis including tumors of any size and stage, LC was comparable to ours (86.5 % and 82.4 %at one and two years) [29].

BED _10_ ≥ 100 Gy is associated with significant higher LC (91.1 % [95 %CI: 81.1;95.9] vs 67.8 % [95 %CI:40.8;84.5]; p = 0.033). This relationship was previously reported by Su et al. [30], highlighting the importance of receiving BED_10_ ≥ 100 Gy in order to improve efficacy (OS, PFS and LC). Fractionations and doses have been very heterogeneous to date, as demonstrated in the recent metanalysis of Bae et al. [31]. The optimal dose in liver SBRT remains a matter of debate. A lot contradictory data exists and recommendations that warrant caution with dose escalation [19], [20].

The main adverse events in our patients were damage to the gastrointestinal and biliary tracts, liver parenchyma injuries and acute general toxicities (Table 4). Although severe, ulcers, fistulas and digestive perforations are rare complications of SBRT, as they represent < 5 % of all events in most prospective studies [32], [33], [34], [35], [36], [37]. Their occurrence generally requires hospitalization with standard medical care, but they can lead to death from hemorrhage or peritonitis. Such vulnerable patients should be followed up by specialized physicians trained to manage liver failure and able to identify potential complications as early as possible. Biliary tract damage is less critical, as abscesses or cholecystitis can be managed by surgery or medical care. Two patients died prematurely from liver failure, despite a restrictive application of dose constraints to the liver. Both had very different disease profiles, with the only common point being that they had received one or more treatments prior to SBRT. These events may have been due to preexisting severe liver damage (induced by chronic liver disease or previous/future treatments), and the implication of SBRT remains difficult to ascertain. Natural history of cirrhosis leads to use of multiple local therapies, with a risk of liver failure. The combination of therapeutic options still needs to be defined, particularly the interval between two local treatments.

Many retrospective studies attempted to compare SBRT to other ablative techniques. Sun et al. and Su et al. compared SBRT with liver resection in a retrospective manner and concluded from retrospective series that SBRT had similar results to surgery with regards to OS and PFS, while treating patients for <5 cm early-stage HCC, with the same profile of toxicity [38], [39]. Studies comparing SBRT to TA reported contradictory findings in metanalysis: Walh et al. reported equivalency of SBRT and RFA in terms of LC and OS, with good tolerance and higher LCs for tumors ≥2 cm after SBRT [40]. Other reports all state this point of view [41], [42]. On the other hand, some metanalysis disprove this finding. For example, Zhang et al. reviewed 8 429 patients from 11 retrospective studies and found better OS in favor of TA, even though LC was higher using SBRT [43]. This difference can be explained by the selection bias given the retrospective nature associated with treatment attribution prioritizing TA for lower stage liver diseases and small size tumors. We can also mention one of the only randomized controlled trial reported by Kim et al. in 2021, comparing TA and hypofractionated radiotherapy, demonstrated the non-inferiority of hypofractionated proton therapy with similar tolerance [44]. The third technique was TACE, commonly used in advanced diseases. Su et al. described significantly better LC using SBRT as first line for BCLC A stage HCC (1-, 3- and 5-years LC: 86,8%, 62,5% and 56.9 %), compared to TACE (1-, 3- and 5-years LC: 69.3 %, 53.3 % and 36.6 %; p = 0.005) [45].

The main challenge relies in combining SBRT with other first-intent treatment to improve the management of liver tumors. In our series, half of the tumors (54.1 %) had already received one or more therapies before receiving SBRT. Prior treatment tended to improve PFS. SBRT can easily be associated with other techniques, and particularly with TACE. In our study, TACE associated with SBRT provided one-year LC of 87.9 % (95 % CI, 72.6 to 94.9). Chemo-embolization alone often allows temporary LC without complete ablation as sterilization of the tumor can be achieved in only 25 % of cases [46]. Thus, the addition of SBRT could improve tumor sterilization in patients for whom TACE alone is insufficient. This association is recommended by numerous authors analyzing therapy by TACE followed by closure SBRT [47], [48], [49]. Nevertheless, a recent trial by Féray et al. in 2023, shows an increase in local control but a higher hepatic toxicity when combining TACE with SBRT [50]. This combination can only be proposed based on the recommendation of an expert center.

Regarding TA, SBRT could serve as a catch-up technique for patients failing first-line ablation, as shown by encouraging data obtained in complex situations with a poor prognosis [51], [52].

SBRT is also substantially beneficial as a bridge therapy in patients awaiting liver transplantation. Eleven of our patients in this situation received bridging SBRT. In the report by Mannina et al. assessing SBRT as a bridge to liver transplantation, 68 % of patients had complete or partial histologic response after radiation therapy, even with suboptimal doses (40 Gy in five fractions for 61 % of them, BED _10_ = 72 Gy) [53]. This option appears to be suitable for intermediate stage disease with larger-sized tumors, for which the current recommended treatment is TACE alone.

There are limitations to the present report in addition to its retrospective nature: the lack of statistical power (with 109 retrospectively analyzed patients), the number of patients assessable for late adverse events (only 45 % had a biological examination to check on potential liver toxicities). Furthermore, patient enrollment led to a selection bias, as SBRT was used as the last option for patients with more complex disease (larger size, unfavorable anatomical situation, following several unsuccessful local therapies) and/or more particular living situation (general health, chronic alcoholism with uncertain alcohol withdrawal, precarity). This bias could result in an underestimation of SBRT's efficacy and an overestimation of its toxicity. Ongoing prospective studies on the role of SBRT will doubtlessly clarify several outstanding issues.

To conclude, our findings demonstrate the efficacy of SBRT for managing patients with primary liver tumors. Its LC rate is similar to that of other local treatments and its safety profile is good in these fragile patients. Given the major comorbidities often associated with this disease, this type of treatment can be administered only in close collaboration with the different medical specialists managing these tumors such as digestive surgeons, radiologists, hepatogastroenterologist-oncologists, and radiotherapists. In addition, the indication must be established by a multidisciplinary tumor board dedicated to the management of these patients. Follow-up must also be multidisciplinary and involve practitioners aware of the main toxicities, which generally occur late in SBRT. SBRT can be considered as a curative alternative for patients ineligible for TA or surgery. Prospective randomized studies are needed to validate SBRT as a standard of care in the same way as other treatments.

## CRediT authorship contribution statement

**Ludovic Hernandez:** Conceptualization, Methodology, Validation, Investigation, Writing – original draft, Visualization. **Laure Parent:** Investigation, Writing – review & editing. **Victoire Molinier:** Conceptualization, Methodology, Investigation. **Bertrand Suc:** Investigation. **Françoise Izar:** Investigation. **Elisabeth Moyal:** Validation. **Jean-Marie Peron:** Investigation. **Philippe Otal:** Investigation. **Amélie Lusque:** Formal analysis, Writing – review & editing. **Anouchka Modesto:** Conceptualization, Validation, Writing – review & editing, Supervision, Investigation, Project administration.

## Declaration of Competing Interest

The authors declare that they have no known competing financial interests or personal relationships that could have appeared to influence the work reported in this paper.
