# Supplementary material for: Stereotactic body radiation therapy in primary liver tumor: Local control, outcomes and toxicities
Source: Clin Transl Radiat Oncol. 2024 Nov 21;50:100892. doi: 10.1016/j.ctro.2024.100892 (PMC11625365; doi:10.1016/j.ctro.2024.100892)
Supplement: Supplementary Data 1 [file mmc1.docx]

SUPPLEMENTARY TABLE: Summary of main prospective and retrospective studies, and meta-analyses (non-exhaustive).

*Biliary Tract Stenosis, perforation or fistula; ** RILD: Radiation-Induced Liver Disease (as specified apart from other “Hepatic failure”); ✢ GDU: Gastric and duodenal Ulcers (as specified apart from other “Gastro-Intestinal Disorders”); ◉ GID: Gastro-Intestinal Disorders (including hemorrhage, perforation, necrosis, nausea, vomiting).

| Type of study | Authors | Year | N° of patients | N° of lesions | Child-Pugh Score - n(%) | BLCL score - n(%) | Mean tumor size in cm (range) | Viral-induced (%) | Mean dose (range) /  Mean fractionation (range) | Mean BED 10 (range) | Mean Follow up in month (range) | Local Control (%)  – at 1,2,3 and 5 years | | | | Overall  Survival (%)  – at 1,2 and 3 years | | | Progression-free Survival (%)  – at 1,2 and 3 years | | | Main reported severe toxicities  (according to CTCAE grading) | |
| --- | --- | --- | --- | --- | --- | --- | --- | --- | --- | --- | --- | --- | --- | --- | --- | --- | --- | --- | --- | --- | --- | --- | --- |
|  |  |  |  |  |  |  |  |  |  |  |  | 1y | 2y | 3y | 5y | 1y | 2y | 3y | 1y | 2y | 3y | Grade 3  Type - % | Grade 4  Type - % |
| Our study | | 2022 | 109 | 118 | A- 84(78)  B- 24 (22) | 0- 7 (6%),  A- 52 (47%)  B- 29 (27%)  C- 21 (19%) | 4.0 (1.6-9) | 20.2 | (30-55) / (3-5) | (48-151) | 22.2(15.1-30.4) | 86.5 | 82.4 | X | X | 83.2 | 73.2 | X | 51.7 | 35.8 | X | *See Table 4.* | |
| Retrospective studies | Jeong et al.[17] | 2018 | 119 | 139 | A- 108 (90.8%)  B- 11 (9.2%) | X | 1.7 (0.8-6.0) | 87.4 | 45 (30-60) / 3 | (60-180) | 25.8 (3.2-36.8) | 98.5 | X | 97 | X | 99.2 | X | 83.8 | 61.5 | X | 33.3 | Biliary tracts* -1.7%  RILD** -1.7% | X |
|  | Teraoka et al.[20] | 2018 | 117 | 144 | A- 103 (88%)  B-14 (12%) | X | 1.6 (1-3) | 88.9 | 48 (40-60) / 5 | (80-120) | X | 100 | 100 | 98 | X | 96 | 81.2 | 65.8 | 50.6-66.5 | X | 26.6-45.3 | X | X |
|  | Sun et al.[21] | 2019 | 108 | 108 | A-108 (100%) | X | 2.3 (0.7-4.9) | 96.3 | 50 (48-54) /  6 (5-8) | 100 (76-102) | 42 (6-77) | 98.1 | 96.2 | 95.1 | X | 96.3 | 89.8 | 80.6 | 85.2 | 70.1 | 60.6 | X | RILD -0.9% |
|  | Scher et al.[18] | 2019 | 136 | 158 | A- 110 (80.9%)  B - 28 (19.1%) | A- 17 (12%)  B- 50 (36%)  C- 71 (52%) | 2.2 (1-7) | X | (45-60) / 3 | (112-180) | 13 | 94.5 | 91 | X | X | 79.8 | 63.5 | X | 61.3 | 39.4 | X | GID◉ -2%  GDU✢ -1%  Hepatic failure -13.7% | GDU -2% |
|  | Roquette et al.[16] | 2022 | 318 | 375 | A- 204 (86.4%)  B- 31 (13.1%)  C- 1 (0.5%) | 0- 33 (12%)  A- 128 (47%)  B- 32 (12%) C/D- 77 (29%) | 3 (5-105) | 12.1 | 45 / 3 | 112 | 70.2 | 97 | 94 | X | 94 | 72 | 44 | X | 62 | 29 | X | Ascites -2.2%  GID -13% | X |
|  | Sanuki et al.[22] | 2014 | 185 | 185 | A- 158 (85.4%)  B- 27 (14.6%) | X | 2.5 | 82.2 | (35-40) / 5 | (59-72) | 24 (3-80) | 99 | 93 | 91 | X | 95 | 83 | 70 | X | X | X | X | Hepatic failure -1.1% |
|  | Que et al.[23] | 2020 | 139 | 221 | A- 122 (87.7%)  B- 17 (12.3%) | X | 5.3 (3.4-8.6) | 87.8 | (26-40) /  (3-5) | 72 (72-85) | 12.8 (1.74-107) | X | 85.1 | X | X | 56 | X | 28 | 35 | X | 14 | RILD -7.9% | Hepatic failure -0.7% |
|  | Munoz-Schuffenegger et al.[24] | 2020 | 128 | 223 | A - 111 (86.7%)  B- 17 (13.3  %) | X | 5.6 (1-20.6) | 68.7 | 33 (27-54) / 5 | (42-102) | X | 87.4 | X | X | X | X | X | X | X | X | X | GID -6.3%  Hepatic failure -3.9% | X |
|  | Matthew et al.[19] | 2020 | 297 | 436 | A- 225 (76%)  B- 65 (20%)  C- 6 (2%) | 0/A-80 (27%)  B- 52(18%)  C- 158 (53%) | 2.7 (0.5-18.1) | 52 | 40 (27-60) /  5 (3-6) | 79 (45-180) | 19.9 | 93.7 | X | X | 86.3 | 77.4 | 39 | 24.1 | X | X | X | Hepatic failure -24.6%  Biliary tracts -1%  Ascites -7.7% | GID -1.3% |
|  | Loi et al.[25] | 2020 | 128 | 217 | A- 92 (72%)  B- 36 (28%) | A- 40 (31%)  B- 72 (56%)  C- 16 (13%) | 3 (1.4-9.9) | 65 | 54 (30-75) /  6 (3-10) | 103 | 19 (3-49) | 87 | 78 | X | X | 83 | 58 | X | 46 | 15 | X | GID -26%  Hepatic failure -8%  Ascites -2% |  |

SUPPLEMENTATY TABLE: *Continuation.*

| Type of study | Authors | Year | N° of patients | N° of lesions | Child-Pugh Score - n(%) | BLCL score - n(%) | Mean tumor size in cm (range) | Viral-induced (%) | Mean dose (range) /  Mean fractionation (range) | Mean BED 10 (range) | Mean Follow up in month (range) | Local Control (%)  – at 1,2,3 and 5 years | | | | Overall Survival(%)  – at 1,2 and 3 years | | | Progression-free Survival(%)  – at 1,2 and 3 years | | | Main reported severe toxicities  (according to CTCAE grading) | |
| --- | --- | --- | --- | --- | --- | --- | --- | --- | --- | --- | --- | --- | --- | --- | --- | --- | --- | --- | --- | --- | --- | --- | --- |
|  |  |  |  |  |  |  |  |  |  |  |  | 1y | 2y | 3y | 5y | 1y | 2y | 3y | 1y | 2y | 3y | Grade 3  Type - % | Grade 4  Type - % |
| Our study | | 2022 | 109 | 118 | A- 84(78)  B- 24 (22) | 0- 7 (6%),  A- 52 (47%)  B- 29 (27%)  C- 21 (19%) | 4.0 (1.6-9) | 20.2 | (30-55) / (3-5) | (48-151) | 22.2(15.1-30.4) | 86.5 | 82.4 | X | X | 83.2 | 73.2 | X | 51.7 | 35.8 | X | *See Table 4.* | |
| Prospective studies |  |  |  |  |  |  |  |  |  |  |  |  |  |  |  |  |  |  |  |  |  |  |  |
|  | Takeda et al.[37] | 2016 | 90 | X | A- 82 (91.1%)  B- 8 (8.9%) | 0- 31 (34%)  A- 45 (50%) B/C- 14 (16%) | X | 89 | (35-40) /  3 | (77-92) | 41.7 (6.8-96.2) | X | X | 96.3 | X | X | X | 66.7 | X | X | 33.9 | Hepatic failure -7.9%  GID -8.9% | X |
|  | Kim et al.[38] | 2018 | 32 | 36 | A- 32 (100%) | A 31 (97%)  C 1 (3%) | 2.1 (1-4.5) | 87.5 | (36-60) /  4 | (68-150) | 27 (12-55) | 90.6 | 80.9 | X | X | 96.9 | 81.3 | X | 62.3 | 42.7 | X | GID -3% | X |
|  | Jang et al.[39] | 2020 | 65 | 73 | A- 64 (98.5%)  B- 1 (1.5%) | 0- 25 (38%)  A- 32 (49%) B/C- 8 (12%) | 2.4 (1-9.9) | 84.6 | (45-60) /  3 | (112-180) | 41 (4-69) | X | 97 | 95 | X | X | 84 | 76 | X | 48 | 36 | RILD -1.5%  GDU -4%  GID -1.5% | X |
|  | Durand-Labrunie et al.[35] | 2019 | 43 | 43 | A- 37 (86.1%)  B- 5 (11.6%) NA- 1 (2.3%) | X | 2.8 (1-6) | 25 | 45 / 3 | 112 | 48 (14.4-55.2) | X | 94 | X | X | X | 69 | X | X | 48 | X | Hepatic failure -13%  Ascites -2% | Hepatic failure -1% |
|  | Bujold et al.[34] | 2013 | 102 | X | A- 102 (100%) | A/B- 35(34%)  C- 67 (66%) | 7.2 (1.4-23.1) | 76.4 | (24-54) / 6 | (33-106) | 31.4 (23.4-36.4) | 87 | X | X | X | 55 | 34 | X | X | X | X | GID -26%  Hepatic failure -1% | GID -2.9%  Biliary tracts -1%  Hepatic failure -4.9% |
|  | Feng et al.[36] | 2018 | 90 | 116 | X | X | 3 (0-13) | 40 | 49 (23-60) / 5 | 98 | 37 | 99 | 95 | X | X | 67 | 36 | X | X | X | X | GID -5.5%  Hepatic failure -14%  Ascites -1.1% | Hepatic failure -7% |
| Meta-analyses | Long et al.[26] | 2021 | 1238 | X | X | X | 2.3 (0.7-6.0) | X | X / X | 100 (59-180) | X | 96 | X | 91 | X | 93 | X | 72 | X | X | X | Hepatic failure -4.3%  RILD -14.7% | X |
|  | Rim et al.[28] | 2019 | 1950 | X | X | X | 3.3 (1.6-8.6) | 76.7 | 83 (48-114) / X | X | X | 85.7 | 83.6 | 83.9 | X | 72.6 | 57.8 | 48.3 | X | X | X | GID -3.9%  Hepatic failure -4.7% | X |
|  | Shanker et al.[27] | 2021 | 2846 | 3088 | X | X | 2.9 (1.6-8.6) | X | 83 (37-137) /  (3-6) | X | X | 91 | 86 | 84 | X | 78 | 61 | 53 | X | X | X | GID -6.5%  Hepatic failure -4% | X |
|  | Bae et al.[30] | 2023 | 1889 | X | A – X (86%) | X | 2.8 (1.3-5.3) | 81 | X | 85.8 (71.4-137.7) | 24 (12-70) | X | X | 81 | 81 | X | X | 64 | X | X | 39 | Nonclassic RILD -8%  Acute grade>3 –(0-30%) | X |
